# Primary Invasive Ductal Carcinoma of the Breast Occurring in a Patient With a History of Hepatocellular Carcinoma Developing From Focal Nodular Hyperplasia: A Case Report and Literature Review

**DOI:** 10.7759/cureus.52818

**Published:** 2024-01-23

**Authors:** Krizia-Ivana T Udquim, Nghi B Lam, Ellery Altshuler, Rick Y Lin

**Affiliations:** 1 Internal Medicine, University of Florida Health, Gainesville, USA; 2 Hematology and Oncology, University of Florida Health, Gainesville, USA; 3 Hematology and Oncology, Johns Hopkins University Department of Medicine, Baltimore, USA

**Keywords:** genetic testing, malignancy, breast cancer, hepatocellular carcinoma, focal nodular hyperplasia

## Abstract

The risk of developing another primary malignancy after an initial liver cancer diagnosis is rare, and the management of multiple primary cancers is not typically discussed. Focal nodular hyperplasia (FNH) is considered a benign tumor, but there have been cases reported that describe hepatocellular carcinoma (HCC) arising from or within FNH. Here, we report a woman in her 70s who had a longstanding history of FNH, later found to be HCC upon resection, who also developed invasive ductal carcinoma. She had no family history of cancer and no genetic testing results were available. Each of her cancers was managed independently, hepatectomy for HCC and neoadjuvant therapy followed by mastectomy for her breast carcinoma. This case demonstrates that the diagnosis of FNH based on radiographic imaging may necessitate a biopsy to confirm diagnosis for a symptomatic patient or those with lesions suspicious for malignancy. It also showcases the importance of close follow-up after a primary cancer diagnosis for the possibility of another primary malignancy emerging. Fresh tissue biopsy for new lesions could help determine primary malignancy or metastasis. Genetic sequencing may help identify driver mutations or genetic alterations that can be targeted.

## Introduction

Hepatocellular carcinoma (HCC) is the fourth most common cancer-related cause of mortality worldwide but accounts for only an estimated 2.2% of all new cancer cases in the United States [1,2]. The typical pathogenesis of HCC involves chronic hepatic inflammation that can lead to cirrhosis and emergence of dysplastic nodules. Genetic alterations can then result in the development of HCC lesions, and a favorable tumor microenvironment eventually can lead to metastatic disease [3,4]. Focal nodular hyperplasia (FNH) is a common benign liver tumor that develops because of increased blood flow to the local hepatic parenchyma [5,6]. Although the transformation of FNH to HCC is very rare, this can occur through the accumulation of genetic aberrations and clonal selection [7]. It is important to note that distinguishing between benign and malignant hepatic lesions can be difficult based on imaging alone [8]. Atypical hepatic lesions may necessitate more than one imaging modality to attain the diagnosis and a biopsy may be needed in a select few circumstances [5]. Another uncommon occurrence is secondary malignancies which have been reported to occur in 2-17% of cases. The frequency of this occurrence depends on the primary cancer type and treatment modalities utilized [9]. Secondary malignancies in patients can occur due to a variety of reasons including genetics, environmental factors, and effects from previous therapies.

## Case presentation

**Figure 1:**
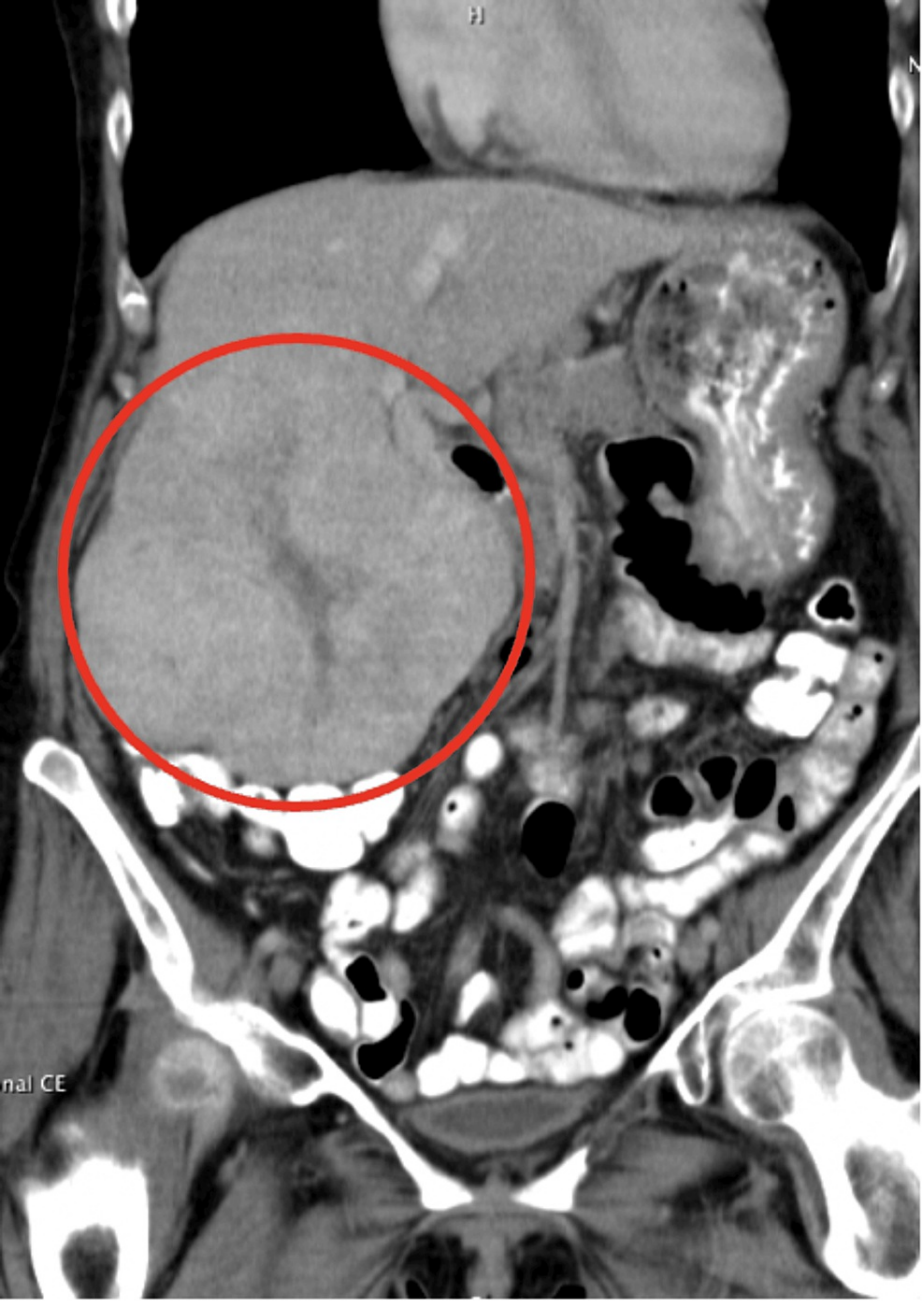
CT scan showing vascular mass in the central portion of the liver 15.5 x 12.0 x 13.5 cm vascular mass denoted with the arrow in the central portion of the liver and involving the right and left hepatic lobes extending inferiorly into the upper pelvis. The lesion demonstrates local mass-effect.

The patient was a female in her 70s with a medical history of chronic obstructive pulmonary disease (COPD), hypertension, and coronary artery disease (CAD) who initially presented in her 50s to an outside facility with symptoms of abdominal pain and weight loss. During that time of initial presentation, she reported being diagnosed with FNH after imaging and a consistent biopsy result of the mass. She was followed with serial computed tomography (CT) scans at outside facilities. In her early 70s, she was referred to our institution after reporting new symptoms of weight loss despite adequate diet intake, daily right upper quadrant pain, ascites, abdominal bloating, fullness, nausea, itching, and malaise. She had no prior history of abdominal surgeries. She was a former smoker who recently quit smoking three years ago and endorsed alcohol use. CT scan of the abdomen and pelvis from the outside hospital showed a vascular hepatic mass in the central portion of the liver involving the right and left hepatic lobes that was exhibiting mass-effect, extending towards the portal region and inferiorly into the pelvis (Figure 1). This hepatic mass was demonstrating mass-effect locally by displacing the gallbladder and biliary tree and was classified as Child-Pugh Class B. Her labs were notable for cancer antigen 19-9 (CA 19-9) at 10.0, carcinoembryonic antigen (CEA) at 5.4, and an alpha-fetoprotein tumor marker (AFP) of 3.9. Her aspartate aminotransferase (AST) and alanine aminotransferase (ALT) were normal at 24 and 19, respectively. Hepatitis A virus IgM antibody and hepatitis C virus antibody were negative.

**Figure 2:**
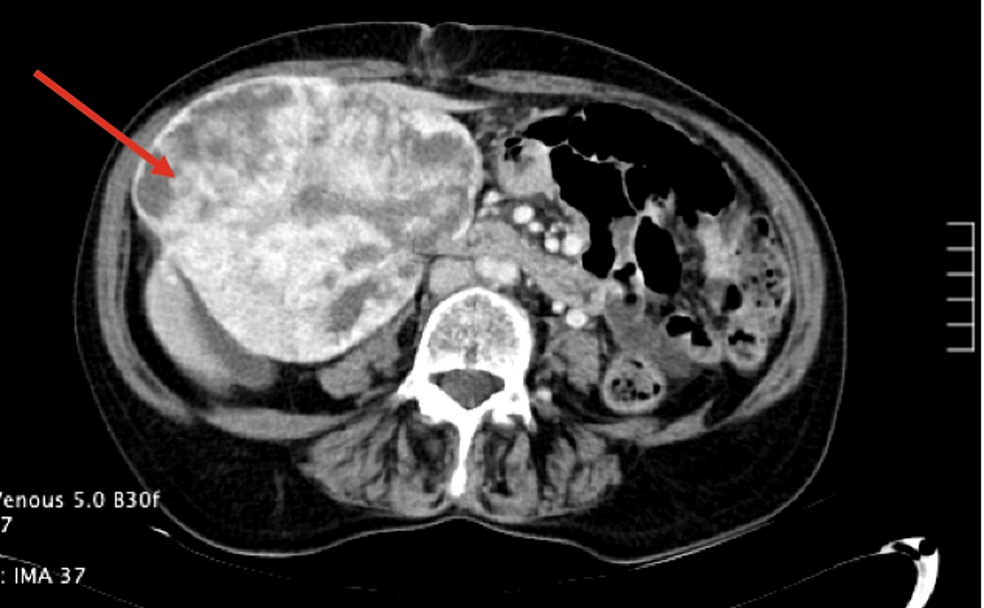
CT scan showing interval stability of the large liver lesion Lesion denoted with the arrow arising from the central liver. Her prior study had described an arterially enhancing liver mass with internal areas of necrosis in the right lobe of the liver consistent with a presumed focal nodular hyperplasia (FNH) with malignant degeneration.

Due to her multiple co-morbidities, she was not initially deemed a surgical candidate (was Barcelona Class B) and received treatment with Yttrium-90. She continued to experience fatigue, poor appetite, nausea with large meals, and dyspnea on exertion. A CT scan of the abdomen, pelvis, and liver showed a lobulated mass in the central liver, exhibiting mass effect on structures on the right side such as the gallbladder, right ureter, and pancreatic head (Figure 2). Since she continued to be symptomatic, she underwent a left portal vein embolization followed by open left extended hepatectomy with portal lymphadenectomy and cholecystectomy. During the open extended left hepatectomy procedure, the mass was identified to be in the middle segment of the liver. An irregular, multinodular mass measuring 13.0 x 13.0 x 9.0 cm was removed. This mass had a central stellate scar and appeared encapsulated and distinct in appearance from the surrounding tissue. Pathology results found it to be well-differentiated T1N0M0 hepatocellular carcinoma (HCC).

**Figure 3:**
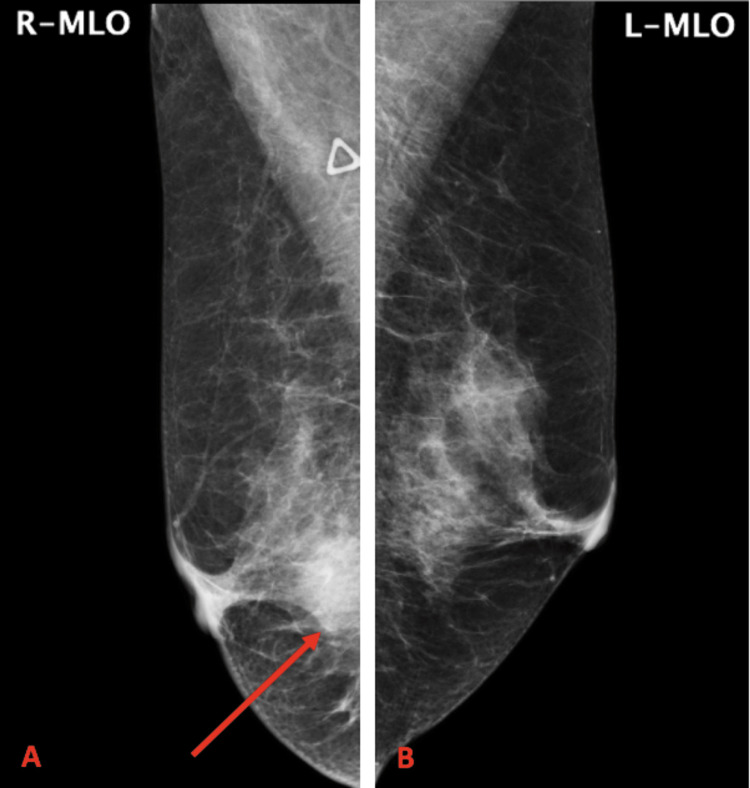
Mediolateral oblique (MLO) mammogram views for her right and left breasts MLO views for the A) right breast showing a dominant 2 cm mass, denoted with arrow, in the central right breast and B) left breast showing no dominant masses or calcifications.

Six months after her resection, the patient presented to an outside facility after she palpated a right axillary lymph node. At the outside facility she was referred for a core needle biopsy of the right axillary lymph node which resulted in positivity for gross cystic disease fluid protein 15 (GCDFP-15), OSCAR cytokeratin, and cytokeratin 7 while weakly positive for mammoglobin. It was negative for estrogen receptor (ER), arginase-1, human melanoma black-45 (HMB-45), paired-box gene 8 (PAX8), cytokeratin 20, caudal type homeobox 2 (CDX2), thyroid transcription factor-1 (TTF-1), and mucin 2 (MUC2). Outside records of the mammogram of her right breast demonstrated that it had heterogenous residual fibroglandular tissue, skin thickening, and partial nipple retraction with a mass in the central retroareolar area measuring 3.0 x 2.2 x 3.4 cm with marked enhancement (Figure 3). Her mammogram was breast-imaging reporting and data system (BI-RADS) V for the right breast.

She had no apparent risk factors for breast cancer and no family history of cancer. Menarche was at age 13 and she was postmenopausal. She was G4P2 with her first pregnancy at 21 years old. She was not on hormone replacement therapy on presentation but reported a history of use in the past. She was not of Ashkenazi Jewish descent. She was referred to our institution where she obtained a core biopsy of the right breast mass which found the tumor to be ER-, progesterone receptor- (PR-), Antigen Kiel 67 (Ki-67)+, and human epidermal growth factor receptor 2 (HER2)+. Positron emission tomography (PET) scan showed a right axillary lymph node with minimal F-fluorodeoxyglucose (FDG) activity (standardized uptake value (SUV) max 1.2) measuring 2.2 x 1.8 cm and a right breast mass measuring 2.7 x 2.1 cm with marked FDG activity (SUV max 16.5). She was treated with six cycles of neoadjuvant therapy comprised of docetaxel, trastuzumab, and pertuzumab prior to surgery. Mammogram after neoadjuvant therapy showed marked response to therapy and resolved right axillary adenopathy. Trastuzumab every three weeks was subsequently initiated and given for ten cycles. She elected for a right-sided mastectomy and sentinel lymph node biopsy. Imaging and biopsy were consistent with T2N1M0 invasive ductal carcinoma of the right breast.

During her course of neoadjuvant therapy, the patient presented to the emergency department several times for dyspnea and was eventually diagnosed with stress cardiomyopathy. Because of her co-morbidities and oxygen dependence, she was not a candidate for radiation treatment for her invasive ductal carcinoma of the breast. The last cycle of trastuzumab was not given due to significant comorbidities and a recent hospitalization. The patient had no evidence of either disease at one year post-treatment.

## Discussion

There have been a few reports of the co-occurrence of HCC with FNH; however, it is rare for HCC to arise within FNH or for HCC to arise from FNH [7,8]. FNH is considered a benign liver tumor that demonstrates a characteristic “central scar” in most lesions [5,7,8]. On CT imaging it displays early contrast enhancement then rapid washout [8]. However, differentiating between benign and malignant hepatic lesions can be difficult using imaging alone [8]. For those with symptomatic or suspicious hepatic lesions such as our patient, tissue biopsy or other surgical interventions should be strongly considered when malignancy cannot be ruled out based on imaging or biopsy [5]. In the case of our patient, the results from her initial diagnosis were self-reported as records were not available.

Ercan et al. reported a case demonstrating a clonal relationship between FNH and HCC following genetic analysis of several lesions from a 74-year-old woman with a history of alcohol abuse and malnutrition who underwent liver tumor resection. They suggested the progression of FNH to HCC after malignant transformation through clonal selection or acquiring additional genetic events [7]. Ercan et al. noted a need to investigate the genomic features of FNH because understanding its progression could help refine surveillance measures for individuals who have these lesions. Haubert et al. reported a case of HCC arising within FNH in an 86-year-old previously healthy woman. Pathology showed that the majority of the mass removed from this 86-year-old was FNH but there were two nodules with characteristics of HCC [8]. This is not the first instance of HCC arising in the background of FNH as Chen et al. reported HCC found partially surrounded by FNH in a 65-year-old woman with a history of pernicious anemia. Chen et al. examined methylation patterns that suggested two different clonal populations [10]. However, Haubert et al. did not do clonal analysis for their patient. They posit though that since the HCC nodules were completely embedded within a background of FNH there could have been a possible malignant transformation [8].

Our patient was found to have a primary liver malignancy, T1N0M0 well-differentiated HCC, around 20 years after her initial diagnosis of FNH. Unfortunately, there were no records available of the tissue biopsy obtained during her initial diagnosis to compare with the results following her tumor resection. Several months later, she was found to have T2N1M0 invasive ductal carcinoma of the right breast. The risk of developing another primary cancer after an initial liver cancer diagnosis was reported to be 1% in 2007 using data from the National Cancer Institute’s Surveillance, Epidemiology, and End Results (SEER) Program database which collected data from 1973-2003 [11]. Common secondary cancers to arise include breast cancer [9,12]. An important part of staging breast cancer is evaluating the axillary lymph nodes which can be done using clinical examination, fine needle aspiration, ultrasound, or core needle biopsy [13]. For our patient, she palpated an axillary lymph node on a self-exam that was later biopsied.

Several factors have been suggested as possible mechanisms for the development of multiple primary cancers, including genetic susceptibility, intensity of previous chemotherapy or radiotherapy, environmental exposure, and specific tumor characteristics [9,12]. The frequency of developing multiple primary cancers has been reported to be between 2-17% [9]. The occurrence of multiple primaries in a single individual can either be synchronous, occurring either simultaneously or within six months of the first malignancy, or metachronous, developing after six months from the first malignancy [9,12,14]. For tumors to be considered separate primary malignancies, they need to arise in different sites and have different histology or morphology to avoid misclassification of metastases or multifocal tumors [9,14]. However, management guidelines for the treatment of multiple primary cancers are lacking [14].

For our patient, each of her primary cancers was treated in isolation with surgical resection for her HCC and neoadjuvant chemotherapy and mastectomy with sentinel lymph node biopsy for her invasive ductal carcinoma. It is important to obtain fresh tissue biopsy for new lesions when determining if the lesion is a primary malignancy or metastasis. Additional research is needed regarding the treatment of patients with multiple primary cancers whether they are synchronous or metachronous [9,12]. These lesions also necessitate the expertise of a multi-disciplinary team to balance the risks and benefits of different treatment modalities for multiple cancers. Next-generation sequencing or genetic studies would have provided insight into any common driver mutations that predisposed her to cancer susceptibility or unique drivers to her tumor histology. A recent American Society of Clinical Oncology (ASCO) provisional clinical opinion recommended using next-generation sequencing for metastatic and advanced solid tumors if there are known FDA-approved biomarker-linked therapeutics for that specific cancer [15].

## Conclusions

To our knowledge, our patient initially diagnosed with FNH and later found to have HCC and subsequent invasive ductal carcinoma of the breast is the first presented. Our patient did not have a family history of cancer nor any genetic testing results available. She did have a long-standing diagnosis of FNH that had been monitored with serial CT scans at an outside facility. Her liver mass had been slow-growing over an extended period; therefore, at the time of initial referral and visit, the concern for malignancy was low. Following her liver resection which diagnosed HCC, she found a palpable axillary lymph node on self-exam that was later biopsied confirming her invasive ductal carcinoma. When treating an initial primary malignancy, it is important to maintain close follow-up for the possible emergence of another primary malignancy at a different site. It is also important to consider obtaining a tissue biopsy for new lesions to determine if the lesion is a primary malignancy or metastasis. Lastly, next-generation sequencing may be beneficial to identify common driver mutations to cancer susceptibility, unique drivers in primary malignancies, or targetable genomic alterations.
